# A portable AFO solution for pneumatic actuator with cable tendon mechanism to assist ankle dorsiflexion

**DOI:** 10.3389/fbioe.2023.1227327

**Published:** 2023-10-19

**Authors:** Junming Wang, Jing Shu, Yujie Su, Chengpeng Hu, Ling-Fung Yeung, Zheng Li, Raymond Kai-Yu Tong

**Affiliations:** ^1^ Department of Biomedical Engineering, The Chinese University of Hong Kong, Shatin, Hong Kong SAR, China; ^2^ Department of Surgery, The Chinese University of Hong Kong, Shatin, Hong Kong SAR, China

**Keywords:** ankle-foot orthosis, pneumatic actuator, stroke rehabilitation, wearable robots, assistive robots

## Abstract

The limited portability of pneumatic pumps presents a challenge for ankle-foot orthosis actuated by pneumatic actuators. The high-pressure requirements and time delay responses of pneumatic actuators necessitate a powerful and large pump, which renders the entire device heavy and inconvenient to carry. In this paper, we propose and validate a concept that enhances portability by employing a slack cable tendon mechanism. By managing slack tension properly, the time delay response problem of pneumatic actuators is eliminated through early triggering, and the system can be effectively controlled to generate the desired force for dorsiflexion assistance. The current portable integration of the system weighs approximately 1.6 kg, with distribution of 0.5 kg actuation part on the shank and 1.1 kg power system on the waist, excluding the battery. A mathematical model is developed to determine the proper triggering time and volumetric flow rate requirements for pump selection. To evaluate the performance of this actuation system and mathematical model, the artificial muscle’s response time and real volumetric flow rate were preliminarily tested with different portable pumps on a healthy participant during treadmill walking at various speeds ranging from 0.5 m/s to 1.75 m/s. Two small pumps, specifically VN-C1 (5.36 L/min, 300 g) and VN-C4 (9.71L/min, 550 g), meet our design criteria, and then tested on three healthy subjects walking at normal speeds of 1 m/s and 1.5 m/s. The kinematic and electromyographic results demonstrate that the device can facilitate ankle dorsiflexion with a portable pump (300–500 g), generating sufficient force to lift up the foot segment, and reducing muscle activity responsible for ankle dorsiflexion during the swing phase by 8% and 10% at normal speeds of 1 m/s and 1.5 m/s respectively. This portable ankle robot, equipped with a compact pump weighing approximately 1.6 kg, holds significant potential for assisting individuals with lower limb weakness in walking, both within their homes and in clinical settings.

## 1 Introduction

After the stroke, the push-off and swing phases of the gait cycle are influenced by impaired paretic ankle function, resulting in reduced forward propulsion symmetry in impaired paretic ankle plantarflexion (PF), poor ground clearance during the swing phase, and uncontrolled landing during weight acceptance in impaired paretic ankle dorsiflexion (DF) (Hall et al., 2011). Foot drop, a common symptom of paretic ankle DF impairment, increases the risk of tripping and falling (de Niet MSc et al., 2008), while foot slap diminishes the desired shock absorption and adds instability to body balance. Ankle-foot orthoses (AFOs) are effective tools for treating ankle paresis caused by muscle weakness, as demonstrated in clinical studies (Takahashi et al., 2015; Zhou et al., 2016; Yeung et al., 2018). Currently, the dominant active AFOs for correcting gait patterns are driven by either electrical motors or pneumatic artificial muscles/fabrics (Awad et al., 2017; Bae et al., 2018; Hong et al., 2018; Yeung et al., 2018; Thalman et al., 2019). Active AFOs with electronic motors have become a popular solution due to their ability to provide sufficient torque for ankle plantarflexion and dorsiflexion assistance. However, conventional rigid electronic AFOs are often designed with rigid structures or components, which add significant inertia to limb movement and may result in misalignment issues (Schiele, 2009; Bae et al., 2015). Moreover, a common issue with electronic motors is their lack of back-drivability, which can make it more challenging for users to perform natural movements (Hussain et al., 2016).

Recent advancements in soft materials and fabrics have led to the development of flexible, lightweight, and ergonomic AFOs in the latest research. Two solutions have been investigated: cable-driven tethered soft AFOs and soft AFOs with fabrics/pneumatic artificial muscles. Bae et al. developed a portable ankle-assisting robot with two electrical motors, which use two Bowden cables to transmit force to the anchor points on the foot to assist plantarflexion and dorsiflexion motion (Awad et al., 2017; Bae et al., 2018; Awad et al., 2020). By aligning with textile suits, the cable-based transmission method resolves the weight problem by tethering Bowden cable from the center trunk to anchoring points at the calf. Other soft AFOs have been developed by utilizing fabrics or pneumatic artificial muscles to decrease mass in the lower limb while providing a large assistance force. For example, Thalman et al. applied a lightweight pneumatic pouch-motor type flat soft actuator on the instep to prevent foot-drop (Thalman et al., 2019). Additionally, Hong et al. utilized a McKibben-type artificial muscle to support swing phase dorsiflexion and a tension spring to support heel rocker function (Hong et al., 2018).

Despite the effectiveness of pneumatic systems in AFOs, their portability is often limited by the high-pressure requirements and time delay responses of pneumatic actuators, which necessitate the use of a powerful and bulky pump, making the entire device inconvenient and heavy to carry. While portable pumps could potentially be installed in a wheeled carrier that automatically follows a walker, the mobility of such a system would still be limited to level ground. In this paper, we propose and validate a concept that enhances portability by employing a slack cable tendon mechanism. By managing slack tension properly, the time delay response problem of pneumatic actuators is eliminated through early triggering, and the system can be effectively controlled to generate the desired force for dorsiflexion assistance.

**FIGURE 1:**
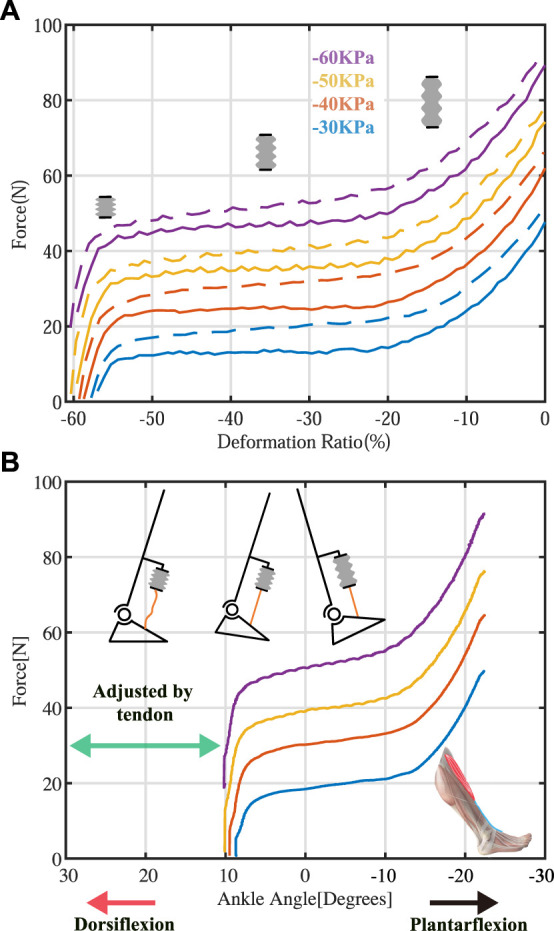
The force-displacement characteristics of the pneumatic actuator **(A)** and the force-angle relationship of the ankle-foot orthosis (AFO) with a pneumatic actuator **(B)**. By adjusting the cable tendon, the onset ankle angles for force initiation can be tuned through the proper tendon length, and the AFO can be operated within the desired angle-force region.

**FIGURE 2:**
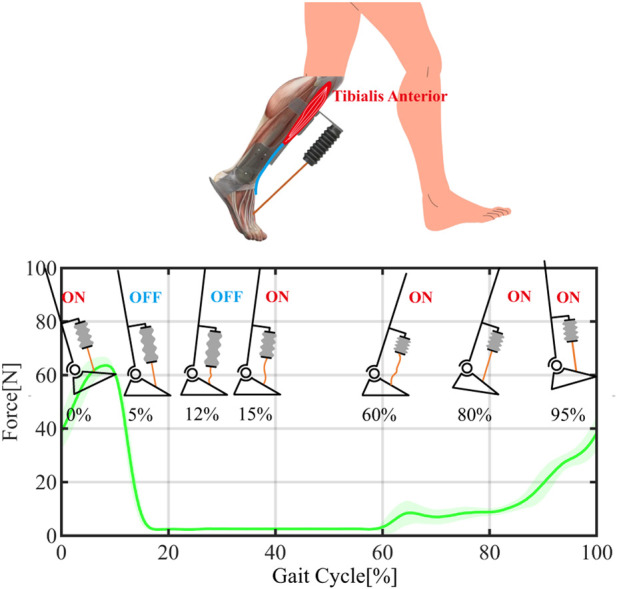
A portable pneumatic AFO with a cable tendon and working mechanism per gait cycle with actuator states. This arrangement allows the system to operate in a simple on/off control manner while compensating for the pneumatic time delay effect. The shadow region represents the 95% confidence intervals.

As shown in Figure 1, the actuation strategy of this AFO can be simplified to on/off control while providing consistent assistance force that intuitively adjusts with the ankle angles in different gait cycles. With proper slack tension management, the AFO induces minimal hindrance to the movement of the ankle, even when triggered during the stance phase, as shown in Figure 2. This minimizes the charge time delay effect of the pneumatic actuator, adding to the potential for a portable solution with a small pump.

This paper presents a portable ankle-foot orthosis (AFO) solution for pneumatic actuators that utilizes slack cable tendon to allow for early triggering of actuation, mitigating the long-time delay effect of pneumatic actuators while causing no hindrance during stance and delivering ankle dorsiflexion assistance during swing and heel landing. To validate its feasibility, a preliminary test was initially carried out on three healthy participants walking on a treadmill. Altogether, this paper contributes:

1. This paper presents the design and characterization of a lightweight and portable ankle-foot orthosis (AFO) solution and cable tendon mechanism that can be adapted to different actuators and adjust to fit the desired ankle angles-force relationship.
2. The proposed solution utilizes a simple on/off actuation strategy that leverages the characteristics of the pneumatic actuator and proper slack tension management to eliminate the charge time delay effect of the pneumatic actuator. This mitigation of the pneumatic time delay effect enables the potential for a portable solution with a small pump.
3. A mathematical model to determine the proper triggering time and volumetric flow rate requirements for pump selection.

We hypothesize that the utilization of a cable tendon mechanism integrated with a pneumatic actuator in an ankle-foot orthosis (AFO) design could compensate for pneumatic time-delay effects, and produce a consistent assistive force that intuitively adjusts with ankle angles in different gait cycles. Additionally, we expect that the integration of this slack cable tendon mechanism with the actuator system requires only a portable small pump to deliver consistent assistive force with improved response to assist ankle dorsiflexion. The subject would require less muscle force from the Tibialis Anterior muscle during walking, and this could be reflected in the EMG signal analysis.

For the following parts of the paper, Section 2 demonstrates the fabrication of the actuator, system overview of AFO with detailed design parameters, and control algorithm. Section 3 presents the necessary experiment setup for determining properties of pneumatic artificial muscle and preliminary walking test. In Section 4, the results of experiments and tests are analyzed and evaluated, while a summary of the current design and future works is discussed in Section 5.

## 2 Design overview and rationale

### 2.1 Design considerations

The design of this AFO aims to provide a lightweight and portable solution that assists ankle DF motion during the swing phase and weight acceptance, with sufficient force for stroke survivors to prevent foot drop and foot slap. Similar to other AFO treadmill walking test (Bae et al., 2015; Awad et al., 2017; Thalman et al., 2019), the subject is asked to walk at the controlled treadmill speed. The AFO was tested on a treadmill with speeds ranging from 0.5 m/s to 1.75 m/s (0.5 m/s, 0.75 m/s, 1.0 m/s, 1.25 m/s, 1.5 m/s, and 1.75 m/s), encompassing the average walking speed of stroke survivors (0.58 m/s; (Wing et al., 2012)) and the average comfortable walking speed for a healthy individual (1.3 m/s; (Bohannon, 1997)). As an essential requirement, the system needs to have a wide range of motion, typically with 20 degrees in dorsiflexion (DF) motion and 30 degrees in plantarflexion (PF) motion (Yeung et al., 2017). Otherwise, it could cause hindrance to the wearer’s normal gait.

In Section 2.2, a 3D-printed vacuum-powered artificial muscle was selected as the pneumatic actuator. In Section 2.3, the structure of AFO and the overall system would be introduced. The control algorithm would be illustrated in Section 2.4.

### 2.2 Design and fabrication of 3D printed vacuum powered artificial muscle

**FIGURE 3:**
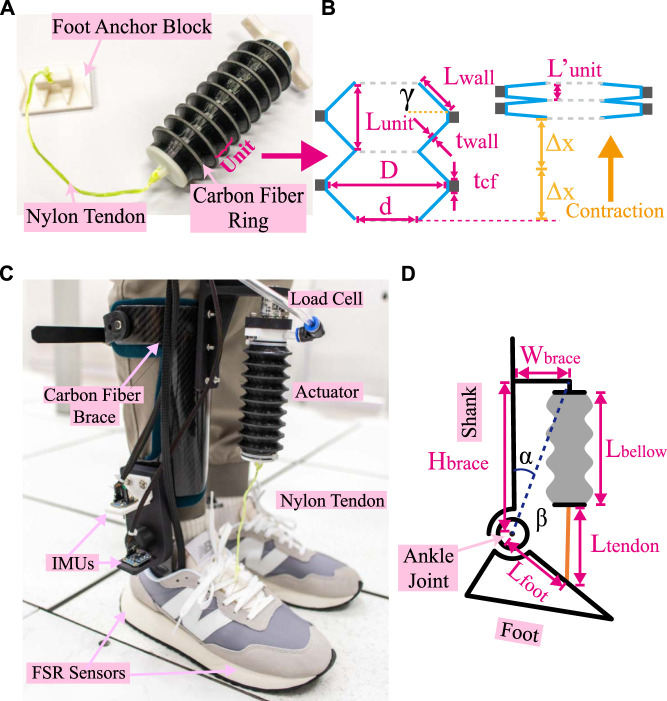
Bellow-shaped artificial muscle. **(A)** Artificial muscle with tendon and foot mounting block. Nine units are contained in the actuator. **(B)** Is the sketch diagram of the cross-section view of two units extracted from **(A)**. In **(B)**, the left figure represents the natural state, and the right represents the compressed state. *L*_*unit*_ and $L_{\mathrm{unit}}^{\prime }$ are the length of units in the natural state and compressed state, respectively. *L*_*wall*_ is the length of the bellow wall. *t*_*wall*_ and *t*_*cf*_ are the thickness of the bellow wall and carbon-fiber ring, respectively. Δ*x* is the contraction length of one unit. *D* and *d* are the outer and inner diameters of the bellow actuator, respectively. **(C)** Structure of Ankle Foot Orthosis for the experiment. The AFO was combined with a carbon fiber brace and a bellow-shaped artificial muscle. Two FSR sensors are embedded in the bottom of the carbon fiber foot brace. Two IMUs near the rotational joint were employed to monitor the ankle angle. A load cell mounted on the actuator base was used to detect the force generated by artificial muscles during walking. **(D)** Kinematic description of the AFO.

As shown in Figure 3, a bellow-shaped artificial muscle was designed, consisting of two main parts: 1) an active contraction actuator in the middle and 2) a non-stretchable tendon at the end of the contraction actuator. The active contraction actuator was 3D printed using TPU filaments with 85A shore hardness (TPU85A, CC3D, China) on a customized FDM 3D printer. Carbon fiber rings with a thickness of 2 mm were CNC-machined and attached to the ridges of the bellow outer shell to increase the artificial muscle stiffness in the radial direction (Figure 3A). With the carbon fiber rings, the actuator wall angle *γ* could be increased to achieve a larger contraction ratio. In this design, the actuator wall angle was set to 45 degrees, which is a balancing point between contraction ratio and contraction resistance force. Two rigid PLA 3D-printed caps were used to seal the artificial muscle with silicon adhesive (Sil-Poxy, Smooth-On, USA). A PU air tube with a 5 mm inner diameter (TU0805, SMC, Japan) was inserted into the actuator through the top cap to extract the air, as illustrated in Figure 3A. A non-stretchable nylon tendon was used to transfer tensile force.

For the contraction ratio bellow-shaped actuator, two units are extracted from Figure 3A, and the sketch diagram of section view is drawn (Figure 3B). Refer to Figure 3B, the length of one unit *L*_*unit*_ in a natural state could be calculated as

$$L_{\mathrm{unit}}=2L_{\mathrm{wall}}\cdot \sin\gamma +t_{cf}$$ (1)

Where *L*_*wall*_ is the length of bellow walls in cross-section view, *γ* is bellow wall angle, *t*_*cf*_ is the thickness of carbon-fiber rings. When the actuator is fully contracted, since the neighbor bellow walls are closely attached, the unit length $L_{\mathrm{unit}}^{\prime }$ could be calculated as

$$L_{\mathrm{unit}}^{\prime }=2t_{\mathrm{wall}}+t_{cf}$$ (2)

where *t*_*wall*_ is the wall thickness of the bellow-shaped actuator. Therefore, for the maximum contraction ratio *η*, we have

$$\begin{array}{ll} \eta & =\frac{\Delta x}{L_{\mathrm{unit}}}\\ & =\frac{L_{\mathrm{unit}}-L_{\mathrm{unit}}^{\prime }}{L_{\mathrm{unit}}}\\ & =\frac{2\left(L_{\mathrm{wall}}\cdot \sin\gamma -t_{\mathrm{wall}}\right)}{2L_{\mathrm{wall}}\cdot \sin\gamma +t_{cf}} \end{array}$$ (3)

Where Δ*x* is the contraction length of one unit. With our design parameters, the bellow-shaped actuator we employed would have a maximum contraction ratio of 60.7%.

### 2.3 Structure of AFO and servo system

This part will illustrate the structure of AFO and its servo system. As shown in Figure 3C, the whole AFO system was combined with 1) a carbon fiber AFO with a rotary shift, 2) a 3D printed pneumatic actuator, and 3) a shoe mounting block. The carbon fiber AFO was similar to the walking robot in (Yeung et al., 2021). Electric servo motors and transmission gears in (Yeung et al., 2021) were replaced with 3D-printed nylon boards to balance the mechanical strength and weight requirements. An L-shaped 3D printed nylon base connected carbon fiber (CF) AFO and artificial muscle. The lower end of the pneumatic actuator was connected to a 3D-printed shoe base with a nylon tendon. With such a design, users can avoid drilling holes in shoes or wearing special shoes.

For the servo system, A vacuum pump with a 50L gas tank (1550D, FUJIWARA, China) was employed for the pneumatic circuit to generate negative pressure for controlled volumetric flow rate test, and six types of small size vacuum pumps were selected for portable solution as detailed in Table1. To minimize the response time of the pneumatic actuator, a high flow-rate 3/2-way pneumatic solenoid valve (VT317V, SMC, Japan) was employed to activate the actuator. A valve driver module (L298N, STMicroelectronics, Switzerland) was used to trigger the solenoid valve. The air pressure inside the actuator chamber was measured by a pressure sensor (XGZP6847A, CFsensors, China). Besides, The AFO is controlled by the Mega 2,560 Pro MCU with the ATmega2560 chip (Arduino, Italy), connected to a PC to transmit data. IMUs (BNO055, Adfruit, USA) located on shank brace and foot brace are used to detect ankle angle. Two insole FSR sensors (MD30-60, K-CUT, China), which can collect dynamic or static force changes in the insole, are located on the bottom of the foot brace to detect human intention during walking. A load cell (SBT671, Simbatouch Electronics, China) was placed on the top of the pneumatic actuator to evaluate the assistance force generated by the artificial muscle during walking.

**TABLE 1:** Technical specification of the portable pump.

Pump model	Operation voltage(V)	Power(W)	Flow Rate(L/min)	Max Pressure(KPa)	Weight(g)
G60-70	24	40	60	−70	550
VN-C4	12	42	40	−85	550
VN-C1	12	10	15	−80	300
HLVP15-C124	24	10	11	−82	360
VN-T1	12	10	10	−81	310
KVP8 PLUSKDS	12	9	6.6	−90	270

**FIGURE 4:**
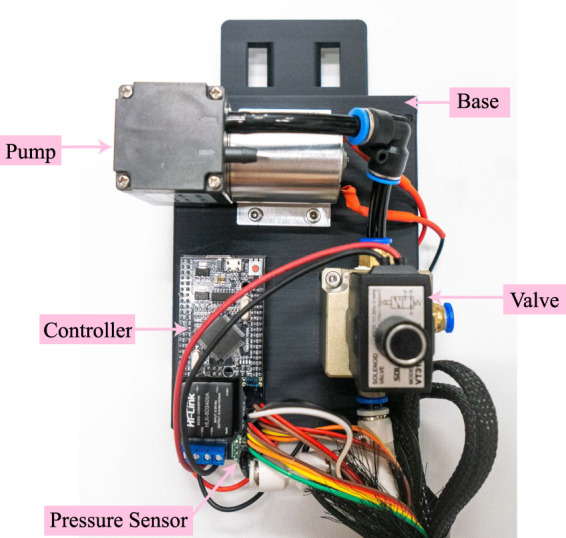
Integration base for the power system. The power system base is worn on the waist belt and houses the pump, valve, controller, and pressure sensor, which are integrated into the front side of the base. The battery and valve driver module are located at the back of the base.

The weight of the pneumatic actuator is 29.7 grams, while the total weight of the actuation system, inclusive of a 0.1 kg load cell, is 0.6 kg when worn at the shank. The weight of the actuation system excluding pump and battery is 750 g. To optimize the weight distribution of the portable version, the heaviest components, including the battery, pump, and valve, are integrated into a base that is capable of wearing on a waist belt as shown in Figure 4.

### 2.4 Control algorithm

The control algorithm for the AFO system can be broadly categorized into three main types: (a) position control, (b) Assist-as-Needed (ANN) control, and (c) force control (Al-Rahmani et al., 2022). In position control, a trajectory generator is used in conjunction with a modern controller to enable the AFO to track the generated trajectory. Conversely, the goal of force control is to maintain a desired constant force output. The ANN control method employs an adaptive impedance control algorithm to modify the assistant force, thereby allowing it to adapt to different levels of disability. This approach is primarily used in robot-aided rehabilitation or training. However, due to the significant time delay required for the pneumatic actuator to attain the desired pressure level, it can only achieve simple on/off control. Additionally, this method is computationally intensive and necessitates repeated fine-tuning of multiple control parameters.

To mitigate these effects, we propose a concise and efficient actuation strategy that delivers consistent force throughout gait cycles that are naturally influenced by ankle angles, by leveraging the actuator’s response and force characteristics and adopting appropriate slack tendon management. The slack tendon management approach enables the actuator to be pre-charged, thereby eliminating the time delay effect, while simultaneously allowing for unrestricted ankle moments during instances where assistance is not required.

**FIGURE 5:**
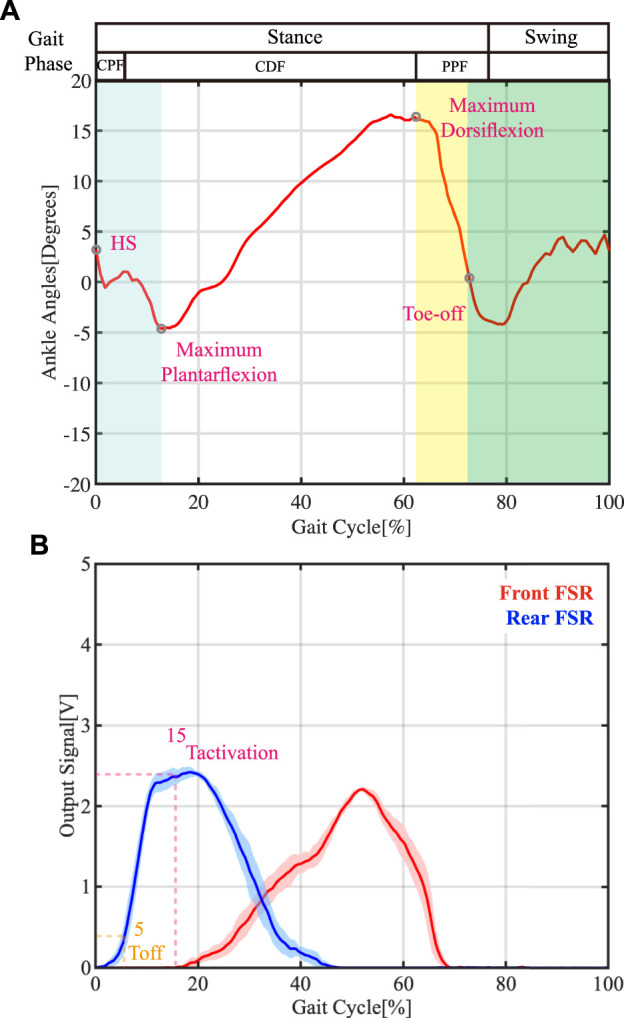
Ankle angle during the gait cycle and FSR sensor readings per gait phase (walking speed: 1.0 m/s). **(A)** Ankle angle of healthy individual. Different shaded colors represent different gait phases. CPF = controlled plantarflexion, CDF = controlled dorsiflexion, PPF = powered plantarflexion, HS = heel strike. **(B)** Shows the reading from the frontal FSR sensor and the rear FSR sensor of healthy subject (*T*_*activation*_ and *T*_*off*_ represent when the actuator was triggered ON and triggered Off, respectively).

According to descriptions in (Perry and Burnfield, 2010; Safaeepour et al., 2014; Van Der Wilk et al., 2018), the gait cycle can be divided into two distinct phases: the stance phase (from heel strike to toe-off) and the swing phase (Figure 5A). As illustrated in the working mechanism of the AFO and actuator states per gait cycle (Figures 1, 2), the actuator is triggered and contracted upon landing, while no force is delivered to the ankle due to the slack tendon. As the ankle transitions into the swing phase, the tendon becomes taut as the foot drops, and the actuator begins providing assistance force to lift the ankle, thus overcoming foot slap. At the heel strike, the assistance force gradually increases at the end of the swing to counteract the deceleration of the foot, and the actuator is gradually released to enable smooth foot landing in the stance phase. By maintaining proper slack tension, the actuator can assist ankle dorsiflexion during the swing phase and foot landing, thereby delivering consistent force assistance at the appropriate time.

In our control algorithm, we incorporate the delay time *t*_*delay*_ (i.e., the time taken for the triggering signal to generate the desired force) as a design parameter. Assuming that the target time step is *T*_*target*_, the triggering time step *T*_*activation*_ can be calculated using the following equation:

$$T_{\mathrm{activation}}=T_{\mathrm{target}}-t_{\mathrm{delay}}$$ (4)

In our system, the AFO is designed to provide sufficient assistance force during the swing phase, which is equivalent to 85% of the gait cycle for the latest target time *T*_*target*_ for the actuator to reach −30KPa, based on the experiment results outlined in Section 4 (details are provided in Section 4.2). After determining the delay time required to reach the desired pressure level (*t*_*delay*_), the corresponding triggering time step *T*_*activation*_ can be computed. To evaluate the feasibility of early triggering times for our AFO, we set *T*_*activation*_ to be 15%. By combining this with the determined *T*_*target*_ (85%), the precise time for *t*_*delay*_ at different walking speeds can be calculated using Equation 4 and presented in Table 2. To buffer the foot landing after the heel strike, the actuator’s force cannot be released immediately, and therefore an off-timing of 5% of the gait cycle, marked as *T*_*off*_ in Figures 5B, C, was set.

**TABLE 2:** Least delay time and required average volumetric flowrate under different walking speeds.

Walking Speed[m/s]	*t*_*delay*_ [s]	Required *Q*_*average*_ [L/min]
0.50	1.40	3.51
0.75	0.93	5.27
1.00	0.70	7.02
1.25	0.56	8.78
1.50	0.47	10.53
1.75	0.40	12.29

In addition, a relevant parameter that can aid in the selection of the appropriate pump is the average volumetric flow rate (*Q*_*average*_), which is a key performance factor of the pneumatic pump that influences the response time of the actuator. The necessary parameter can be determined by the changing volume of the actuator (*V*_*change*_) and the delay time required to reach the desired pressure level (*t*_*delay*_)

$$\frac{V_{\mathrm{change}}}{t_{\mathrm{delay}}}=Q_{\mathrm{average}}$$ (5)

Subsequently, the corresponding requirement for the average volumetric flow rate (*Q*_*average*_) can be calculated for different walking speeds using Equation 5, as summarized in Table 2.

## 3 Experiment setup

### 3.1 Benchtop experiment setup

The characteristics of the pneumatic actuator were determined through benchtop experiments. Given the presence of a flexible TPU material and bellow-shaped structure, the force output of this type of actuator depends on the internal pressure and contraction ratio (Chen et al., 2018), which can be characterized by the force-length relationship that is similar to that of skeletal muscles (Kotak et al., 2022). The two ends of the artificial muscle were fixed onto a customized universal tensile test machine. To measure the generated force and displacement of the artificial muscle, a load cell (SBT671, Simbatouch, China) and a rope encoder (MPS-XS-1000, Miran, China) were utilized, respectively. Data was recorded and transmitted to a computer using a DAQ card (USB6212, NI, USA), with the collected data being analyzed using Matlab software (R2020b, The Mathworks, USA).

To determine the relationship between output force and displacement of the bellow-shaped actuator, the lower end of the artificial muscle was fixed and connected to the load cell. The upper end of the artificial muscle was driven up and down by a vertically moving slide with a constant moving speed (1.63 mm/s, quasi-static motion). A proportional valve (ITV 2090, SMC, Japan) was employed to maintain the pressure inside the artificial muscle chamber at a constant pressure setting value (−30, −40, −50, −60 kPa). The displacement of the upper end of the actuator was measured using a rope encoder.

To determine the artificial muscle’s response time and real volumetric flow rate using a small portable pump, only one end of the artificial muscle was fixed. The chamber of the artificial muscle was connected to the vacuum pump via a solenoid valve, and the length of the PU tube was set to 0.6 m, which is the same as the setting in the walking test. At the start of the experiment, the solenoid valve would be triggered on when *t* = 0.5 s and triggered off when *t* = 4.0 s. The pressure value inside the chamber and volumetric flow rate in the first 5 s were recorded by the DAQ device, as mentioned above. The actual volumetric flow rate was measured using a digital flow switch (PFM711-02-C, SMC, Japan). To test the feasibility of the portable solution, six small pumps were selected and tested to determine the corresponding response time and real volumetric flow rate. Details of the device are provided in Table 1.

### 3.2 Walking test setup

To verify the feasibility of the system, a preliminary test was conducted on a healthy male participant (Subject 1, age: 27, height: 1.76 m, weight: 60 kg) who walked on a treadmill (iRun 4.0, Reebok, UK). Surface electromyography (sEMG) sensors (Delsys Trigno, Delsys, Natick, MA) were utilized to monitor the muscle activity of the participant’s tibialis anterior muscle. The participant was instructed to walk on the treadmill at five different speeds (0.5 m/s, 0.75 m/s, 1.0 m/s, 1.25 m/s, 1.50 m/s, and 1.75 m/s). The AFO was considered to be working effectively if the participant’s muscle effort of tibialis anterior during the swing phase and loading response phase was reduced. The maximum voluntary contraction (MVC) of the tibialis anterior muscle was recorded before the start of testing, with the participant exerting maximum dorsiflexion effort against a fixed surface while sitting in a chair. The muscle activity of the tibialis anterior muscle at rest was measured as well, prior to MVC test. The human subject experiment was approved by the Joint Chinese University of Hong Kong-New Territories East Cluster Clinical Research Ethics Committee (2018.493), and the trials were registered at ClinicalTrials.gov with registration number NCT02471248.

#### 3.2.1 Preliminary tests with controlled flowrate via big pump

##### 3.2.1.1 Test #1: preliminary test without AFO

Prior to wearing the AFO, the healthy subject was required to walk on the treadmill without wearing it. To assess the subject’s muscle effort at different walking speeds, the treadmill speed was gradually increased to the desired testing speed, with an adaptation time of 1 min, and a total walking time of 4 min was set, which is similar as settings in (Thalman et al., 2021). The healthy subject was given an 8-min rest period between each test. Previous studies of AFO on treadmill walking tests have shown that the resting period ranges from 5 to 10 min (Kim et al., 2017; Kim et al., 2020). The 8-min rest period provides sufficient time for the muscles to recover without significantly increasing the overall duration of the testing session. The muscle effort was recorded using sEMG sensors.

##### 3.2.1.2 Test #2: FSR sensor triggering threshold value determination

To determine the threshold value of FSR sensor triggering, the participant was asked to wear the AFO and walk on the treadmill (without actuator activation) at a controlled speed of 1.0 m/s. The ankle angle was recorded using IMUs on the AFO, and the threshold value for FSR sensor triggering was subsequently identified.

##### 3.2.1.3 Test #3: AFO performance evaluation with controlled pressure and volumetric flow rate

To evaluate the performance of the AFO, the participants were required to wear it and walk on a treadmill with actuator activation at different speeds. To initially verify the feasibility of the portable solution, the output pressure from the large vacuum pump was restricted to −60 kPa. Additionally, the volumetric flow rate was controlled to mimic the real parameter of a small-sized portable pump, ranging from 16 L/min to 7 L/min when opening to air, as detailed in Table 3. The air tube length from the solenoid valve to the actuator was 0.6 m. The force generated by the artificial muscle was recorded by the load cell with a frequency of 20 Hz, while the frequency of IMU recording was 100 Hz.

**TABLE 3:** Setting of volumetric flow rate under pressure of −60 kPa with big pump.

Open volumetric flow Rate[L/min]	Connected volumetric flow Rate[L/min]
16	13
13	10.8
10	8.85
7	6.53

#### 3.2.2 Preliminary tests with portable pump

The participant also walked on the treadmill, with the setup being identical to the test in Section 3.2.1.3, except for the use of selected portable pumps and the corresponding supported treadmill speed. And another two healthy male participants (Subject 2, age: 26, height: 1.80 m, weight: 90 kg, and Subject 3, age: 23, height: 1.85 m, weight: 64 kg) were also invited to validate the portable solutions.

## 4 Experiment results

### 4.1 Characteristics of 3D printed vacuum powered artificial muscle

#### 4.1.1 Actuator force generation versus displacement

**FIGURE 6:**
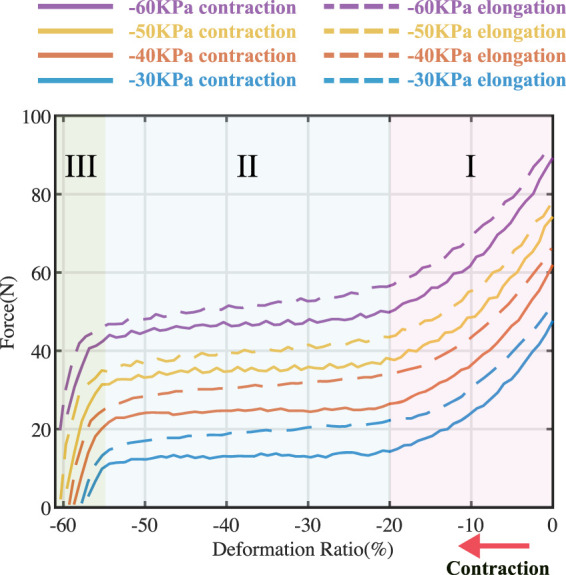
Force-displacement test. The line with different colors illustrates the relationship of the actuator under different pressure, ranging from −30 kPa, −40 kPa, −50 kPa, and −60 kPa, respectively. Solid lines and dashed lines represent when displacement increased and decreased, respectively. The output force of the actuator could be divided into three stages based on the slope of the curves.

Figure 6 illustrates the relationship between generated force and displacement of the bellow-shaped actuator, with the inside pressure varying between −30, −40, −50, and −60 kPa. The solid lines and dashed lines represent when the bellow was shortened (displacement increased) and elongated (displacement decreased), respectively. Generally, the force output positively increased with the supplying pressure.

The force output of the actuator could be categorized into three stages, as determined by the slope of the curves. In stage I, the output dropped linearly as the displacement Δ*L* increased until 20%. Then, the output force reached a plateau stage (stage II in Figure 6), with Δ*L* increasing until 55%. Therefore, when the pressure inside the actuator chamber is constant, there is a ‘quasi-constant’ region in force output when Δ*L* ∈ {20%,55%}. In stage III, the generated force dropped linearly (from 50% of the maximum block force to zero) with a rapid slope until the bellow-shaped actuator was fully contracted, while the displacement was only 5%.

A proper slack tendon mechanism enables a simple and compact actuation strategy (on/off control). By adjusting the length of the nylon tendon, the actuator can operate in stage I and stage II during ankle dorsiflexion (as shown in Figure 1), generating assistance force that intuitively adjusts with ankle angles. This method ensures the consistency of assistance force in different gait cycles, while the slack tension before the swing motion allows no hindrance to be delivered to the ankle, even when the actuator is triggered. Thus, the time delay effect of the pneumatic actuator can be mitigated by early triggering.

#### 4.1.2 Step response of bellow-shaped actuator

As illustrated in Figure 1, the slack tension before the swing motion allows for more charge time to compensate for the long time delay effect of the pneumatic actuators, which has the potential for a portable solution using a small pump. The response time for using small pumps is listed in Table 4. According to the estimated allowable delay time in Table 2, powerful pumps such as the G6070 or VN-C4 could have a high volumetric flow rate and low response time that could potentially support high walking speeds up to 1.75 m/s. However, small pump like KVP8 PLUSKDS with low volumetric flow rate of 3.18 L/min could not meet the requirement for the lowest walking speed (3.53 L/min for 0.5 m/s). Nonetheless, the VN-C1 pump could support walking speeds up to 1 m/s due to its low response time, even though its average volumetric flow rate is lower than the corresponding required volumetric flow rate (7.02 L/min for 1 m/s in Table 2).

**TABLE 4:** The step response of the actuator with small pumps.

Pump model	*t*_*delay*_ −30 KPa[s]	*t*_*delay*_ −40 KPa[s]	*t*_*delay*_ −50 KPa[s]	Average Q to *V*_*Change*_[L/min]	Open Q[L/min]
G6070	0.162	0.381	0.455	14.19	19.79
VNC4	0.241	0.467	0.555	9.71	20.12
VNC1	0.716	0.862	1.029	5.36	10.20
HLVP15C124	1.381	1.653	1.894	4.14	7.27
VNT1	1.407	1.681	1.972	4.03	7.34
KVP8 PLUSKDS	1.660	1.951	2.190	3.18	5.02

Within this estimated allowable delay time, a small portable pump with a high flow rate such as pump models G60-70, VN-C1, or VN-C4 is preferred. We used the VN-C1 or VN-C4 pump for the feasibility test of a portable AFO. Within this portable solution, the wearable weight at the trunk (waist) would be 1.06 kg, and the overall weight could be controlled within 1.6 kg excluding the battery. Kim et al. (2017) developed a portable pneumatic AFO using a miniature custom compressor for drop foot correction, which weighed 3.6 kg excluding the battery. Our method reduces the requirement for a pump, thereby greatly reducing the total mass of the portable system.

### 4.2 Walking test results

#### 4.2.1 FSR reading

The reading of FSR sensors in the gait cycle of the assisted side of the participant is shown in Figure 5B. As shown in Figure 5A, the stance phase represents the period when there is contact between foot and ground (i.e., from heal strike state to toe-off state). The stance phase could be further divided into three sub-phases based on the ankle angle changes: 1) controlled plantarflexion (CPF) phase: from heel strike to maximum plantarflexion. 2) controlled dorsiflexion (CDF) phase: from maximum plantarflexion to maximum dorsiflexion. 3) powered plantarflexion (PPF) phase: from maximum dorsiflexion to toe-off state. In the CDF phase (from maximum plantarflexion to maximum dorsiflexion), the FSR reading from the frontal insole increased rapidly, while the FSR reading from the rear insole gradually dropped to zero. Such periodic changes in insole dynamic force can help determine the necessary parameters in our control algorithm.

By fine-tuning the length of the nylon tendon, the triggering time step can be advanced to the CDF phase region, thanks to the slack tendon. At the start of this gait cycle phase, the FSR reading from the rear insole gradually reduced from its peak, while the frontal FSR reading started to increase, as shown in Figure 5B. Therefore, a threshold triggering value can be set to balance the assisting effects of the AFO and the reliability of the actuator triggering mechanism. To test the feasibility of our AFO on early triggering time, we set *T*_*activation*_ to 15% of the gait cycle. To buffer the foot landing after the heel strike, the force of the actuator cannot be released immediately. Thus, the off timing is set to 5% of the gait cycle, marked as *T*_*off*_ in Figure 5B.

#### 4.2.2 AFO performance evaluation with controlled volumetric flow rate

**FIGURE 7:**
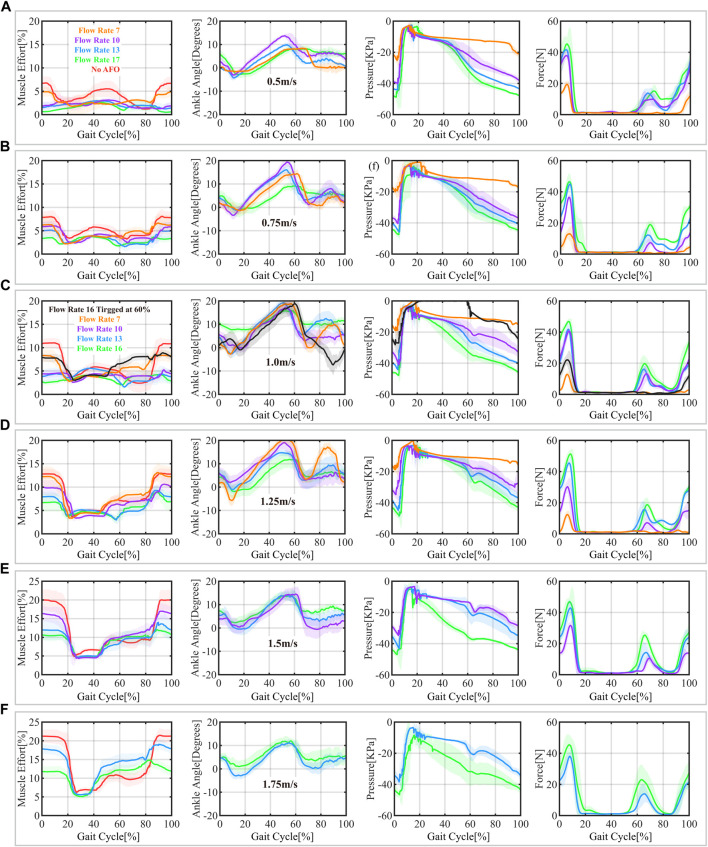
Status of the AFO during the gait cycle, including sEMG data normalized over MVC for the tibialis anterior muscle, ankle trajectories (ankle joint angle), and sensor measurements (pressure inside actuator chamber and assistive force generated by the pneumatic actuator) at different treadmill speeds: 0.5 m/s **(A)**, 0.75 m/s **(B)**, 1 m/s **(C)**, 1.25 m/s **(D)**, 1.5 m/s **(E)**, and 1.75 m/s **(F)**. The red curve represents reference data without the AFO, while the curves with other colors illustrate data with the AFO in different pre-set volumetric flow rates. The shadow region represents the 95% confidence intervals.

The status of the system, which includes the participant’s sEMG data and sensor measurements of the AFO during walking on a treadmill at speeds ranging from 0.5 m/s to 1.75 m/s, is shown in Figures 7A–F. Seven continuous gait cycles were chosen after the adaptation time to reflect the real walking condition and the status of the actuator. This number of cycles allowed us to capture sufficient data to evaluate the performance of the AFO while minimizing the burden on the participants. The results in Figure 7 showed reduced muscle effort with the assistance of the powered ankle-foot orthosis. The muscle effort without the AFO (red curve) was presented as a reference. The AFO with a higher flow rate generated more assistance force and, therefore, reduced more muscle effort, especially at fast treadmill walking speeds (reduced up to 9% of muscle effort at 1.75 m/s). However, the AFO with a lower flow rate than the requirement under a specific walking speed had less or no assistance effect, as predicted in Table 2. For example, the muscle effort of the AFO with a flow rate of 7 L/min under 1.0 m/s was only reduced by 3% since it could not generate enough assistance force. Due to the time delay response, a similar situation occurred at the same treadmill walking speed but with the highest flow rate and triggering at 60%. The results generally corresponded to the estimated average volumetric flow rate (from Table.2) determined from mathematical model.

The pressure and force plots in Figure 7 show that the assistive force is appropriately delivered to the patients at the right time, as DF assistance is effective during the swing and weight acceptance stages, even when triggered early. The assistance force level depends on the pressure inside the actuator. Due to the slack tension, force is not delivered to the ankle even when the inside pressure reaches its maximum. Additionally, the slack tendon allows the AFO to minimize the impedance to natural movement when no assistance is needed at the stance phase, as seen from the minimal force before the swing phase. There is a slight peak force after toe-off, resulting from the angular acceleration of foot in dorsiflexion direction along ankle joint generated by tensioned slacked cable tendon. The assistance force kept increasing at the end of the swing to compensate for the deceleration of the foot after the heel strike, and the actuator released gradually to land the foot smoothly in the stance phase.

#### 4.2.3 AFO performance evaluation with portable pump

**FIGURE 8:**
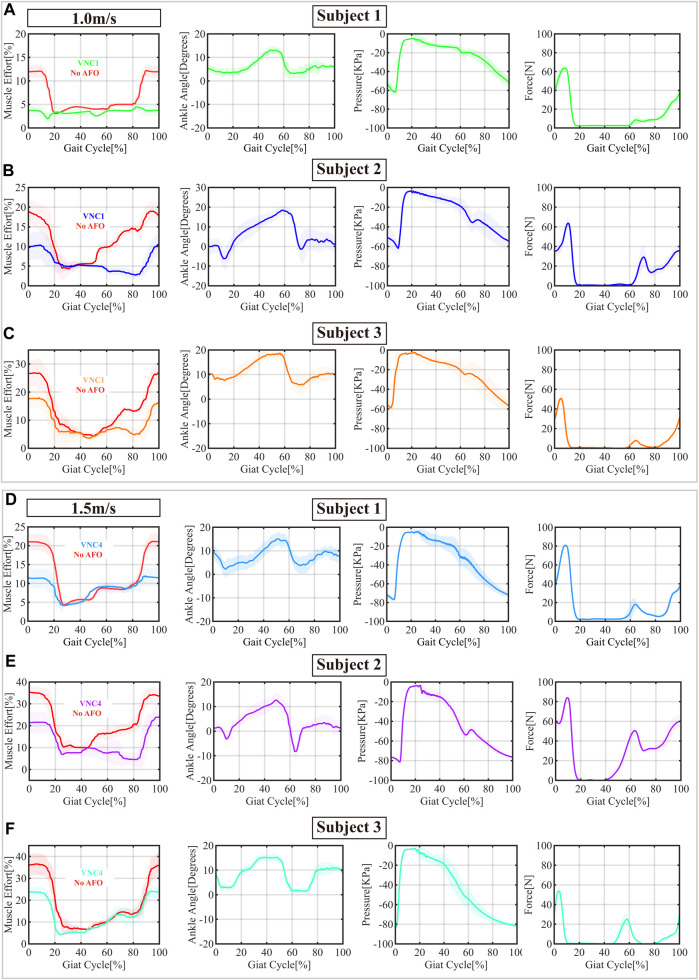
The sensor measurements of three subjects wearing the portable ankle-foot orthosis (AFO) with two portable pumps operating at treadmill speeds of 1.0 m/s **(A–C)** with the VNC1 pump, and 1.5 m/s **(D–F)** with the VNC4 pump, respectively. In the sEMG measurement profile, the red curve represents reference data without the AFO. The shadow region represents the 95% confidence intervals.

Based on the previous step response results and the AFO performance evaluation with controlled flow rate, it is evident that the slack tendon mechanism allows the AFO with controlled flow rate to assist ankle dorsiflexion. In this study, we demonstrate the portable version of the AFO solution using two selected small pumps: VN-C1 and VN-C4. The previous step response results in Table 4 show that the VN-C1 and VN-C4 are capable of supporting walking speeds up to 1 m/s and 1.5 m/s, respectively, according to the estimated least response time and flow rate requirement in Table 2. The sEMG data in Figure 8 shows that both AFOs function well in all three subjects with different walking speeds, as the muscle effort decreased by about 8%, 8% and 9% with VN-C1 in 1 m/s treadmill walking speed, and 9%, 11%, and 12% with VN-C4 in 1.5 m/s treadmill walking speed, respectively. All the ankle angles of three subjects remained above 0 degrees in the swing phase, and a consistent assistance force was delivered to help ankle dorsiflexion and foot landing. And the level of assistance force was adjusted with the ankle angles following the force-angle relationship in Figure 1.

## 5 Conclusion and future work

This paper presents the design, characterization, and evaluation of an ankle-foot orthosis actuated by a pneumatic actuator with a cable tendon mechanism. By utilizing proper tendon slack, the actuation strategy could be simplified to on/off control with compensation for the pneumatic time delay effect while producing consistent assistive force that intuitively adjusts with ankle angles in different gait cycles. The integration of the slack cable tendon mechanism with the actuator system necessitates only a portable small pump for the delivery of reliable assistive force with prompt response, specifically targeting ankle dorsiflexion. The outcomes of pilot human trials demonstrate that the developed system effectively reduces muscle activity responsible for ankle dorsiflexion during normal walking speed, thus providing assistive force. This portable ankle robot with a small pump weighted approximately 1.6 kg. Clinicians have the potential to prescribe this ankle device to assist people with weakness on lower limb for walking at home or in centre. In future investigations, it is intended to conduct additional tests on impaired individuals exhibiting various degrees of dropped foot, aiming to enhance their ankle dorsiflexion through the utilization of the proposed system.

## Data Availability

The original contributions presented in the study are included in the article/Supplementary Material, further inquiries can be directed to the corresponding authors.
